# Novel efficient reservoir computing methodologies for regular and irregular time series classification

**DOI:** 10.1007/s11071-024-10244-3

**Published:** 2024-09-06

**Authors:** Zonglun Li, Andrey Andreev, Alexander Hramov, Oleg Blyuss, Alexey Zaikin

**Affiliations:** 1 Department of Mathematics, University College London, London, UK; 2 Department of Women’s Cancer, Institute for Women’s Health, University College London, London, UK; 3 Baltic Center for Neurotechnology and Artificial Intelligence, Immanuel Kant Baltic Federal University, Aleksandra Nevskogo Str., 14, Kaliningrad, Russia 236041; 4 Wolfson Institute of Population Health, Queen Mary University of London, London, UK; 5 Department of Pediatrics and Pediatric Infectious Diseases,Institute of Child’s Health, Sechenov First Moscow State Medical University,Sechenov University, Moscow, Russia 119991; 6 Britton Chance Center for Biomedical Photonics, Wuhan National Laboratory for Optoelectronics-Huazhong University of Science and Technology, Wuhan, China; 7 Lobachevsky State University of Nizhniy Novgorod, Prospekt Gagarina 23, Nizhniy Novgorod, Russia 603022

**Keywords:** Reservoir computing, Echo state networks, Nonlinear dynamical systems, Time series classification, 34A34, 62P10, 68T07

## Abstract

Time series is a data structure prevalent in a wide range of fields such as healthcare, finance and meteorology. It goes without saying that analyzing time series data holds the key to gaining insight into our day-to-day observations. Among the vast spectrum of time series analysis, time series classification offers the unique opportunity to classify the sequences into their respective categories for the sake of automated detection. To this end, two types of mainstream approaches, recurrent neural networks and distance-based methods, have been commonly employed to address this specific problem. Despite their enormous success, methods like Long Short-Term Memory networks typically require high computational resources. It is largely as a consequence of the nature of backpropagation, driving the search for some backpropagation-free alternatives. Reservoir computing is an instance of recurrent neural networks that is known for its efficiency in processing time series sequences. Therefore, in this article, we will develop two reservoir computing based methods that can effectively deal with regular and irregular time series with minimal computational cost, both while achieving a desirable level of classification accuracy.

## Introduction

Time series refers to a sequence of data points collected in a chronological order over a period of time, with each point typically being recorded at a specific timestamp. A time series has two main components, timestamp and observation. Timestamp presents the time at which a specific record is taken while an observation displays a value associated with each timestamp that informs the relative importance to the other time points. Additionally, time series data may come with some other patterns that make the analysis of time series more challenging. For instance, samples from the same dataset may have different lengths (variable length) and/or adjacent time points may have different time intervals (heterogeneous interval). Time series analysis involves studying and interpreting patterns such as trends and dependency within the sample over time and has been widely applied to real-world phenomena [1–3]. Among them, time series classification (TSC) focuses on the task of categorizing and labeling sequential data into their distinct classes or categories and plays an indispensable role in medicine, telecommunications and finance, etc. The efficacy of TSC algorithms relies on their capability of balancing short- and long-term memory as well as capturing the time dependency, whilst distinguishing desired patterns from the noisy ones.

Over the past few decades, an astronomical amount of algorithms have been developed to address this particular field. Thus far, Long Short-Term Memory (LSTM) networks can be seen as a milestone breakthrough, offering a robust solution to the challenges posed by modelling complex long-term dependencies in sequential data [4–7]. LSTM networks are a type of recurrent neural networks (RNN) that take advantage of memory cells and gates as a means to control the flow of information through the network. The design of the network was largely motivated to mitigate the bottleneck of vanishing gradient. However, the training of the network is enabled by the state-of-the-art backpropagtion through time (BPTT) techniques. While BPTT is a powerful and effective method, it can be computationally expensive, not least for large and deep neural networks. Apart from backpropagation-facilitated neural networks, distance-based methods have also demonstrated enormous success on a wide range of TSC tasks [8–10] and among them, 1-Nearest Neighbour Dynamic Time Warping (1NN-DTW) has been proven difficult to beat as compared to other similar methods [11]. Nevertheless, 1NN-DTW requires the computation of the pairwise distance between samples which can still substantially increase the computation overhead especially when the sample size is large. As a result, the quest for some more efficient methods has never ceased in an attempt to strike the right balance between accuracy and energy consumption.

Complex dynamical systems have demonstrated colossal potential in learning and computation in a wide spectrum of frameworks such as gene regulatory networks, cellular networks and artificial neural networks [12–16]. Among them, reservoir computing (RC) stands at the forefront of cutting-edge research in the field of machine learning and artificial intelligence, providing a promising approach to the challenges of processing complex temporal data [17–19]. It maps input signals to a non-linear high-dimensional dynamical system where neurons are recurrently connected, generating a comprehensive representation of the input features. The training is only applied to the output layer which makes it a hyper-efficient alternative to the mainstream deep neural networks including LSTM networks. Additionally, RC can also be regarded as a mini-brain. It is therefore, a more biologically plausible model and may pave the way for better understanding the information processing in the brain [20–22]. Echo state networks (ESNs) are an instance of RC where sigmoid functions have been employed as the activation functions in the reservoir. They have drawn growing attention over the past few decades by virtue of their ease of implementation and their computational efficiency [23–25]. Nevertheless, most of these methods are focused on sequential predictions and the classification methods are still underdeveloped, partially down to the non-existence of BPTT training. Namely, the loss at the terminal prediction may not be able to flow back and adapt the weights in the previous layer(s). In order to get around this obstacle, some solutions have been proposed over the past decade: [26] employed the idea of time warping invariance presented in [27] and modified the structure to accommodate TSC; [28] mapped the input signals into different state clouds in the reservoir layer and the parameters were optimized with the adaptive differential evolution; [29] substantially enhanced the classification accuracy by leveraging discriminative feature aggregation and outlier-robust weights algorithms to adjust the weights in the input and the output layer, respectively. Nevertheless, despite their tremendous effort, they generally demand the extension to the classic RC structure and additional training algorithms for the input and the output layer. Furthermore, these methods were only tested on regular time series and to the best of our knowledge, most of them are not inherently compatible with the irregular time series data. Hence, in this work, we will propose two new methods for ESNs that can efficiently perform TSC tasks for regular and irregular data, respectively, whilst maintaining a desirable classification accuracy. More importantly, our methods only rely on the fundamental structure of RC, which resembles the way that the brain receives and processes the information. Unfortunately, there does not exist a precise definition for *regular* and *irregular* data. Loosely speaking, in this work, we refer to the sequences from the same dataset as *regular* if they have the same and a sizable length, and the intervals between the adjacent timestamps are even.

The article will be organized as follows: Sect. 2 will introduce two new methodologies that we proposed in the context of ESNs. Section 2.1 provides an overview of the ESNs, Sect. 2.2 presents a novel method that can address a wide range of datasets with regular time series whereas Sect. 2.3 presents an alternative to address the irregular ones. Section 3 reports the performance of our new methods and Sect. 4 includes the conclusion and discussion.

## Methods

### Echo state networks

**Fig. 1:**
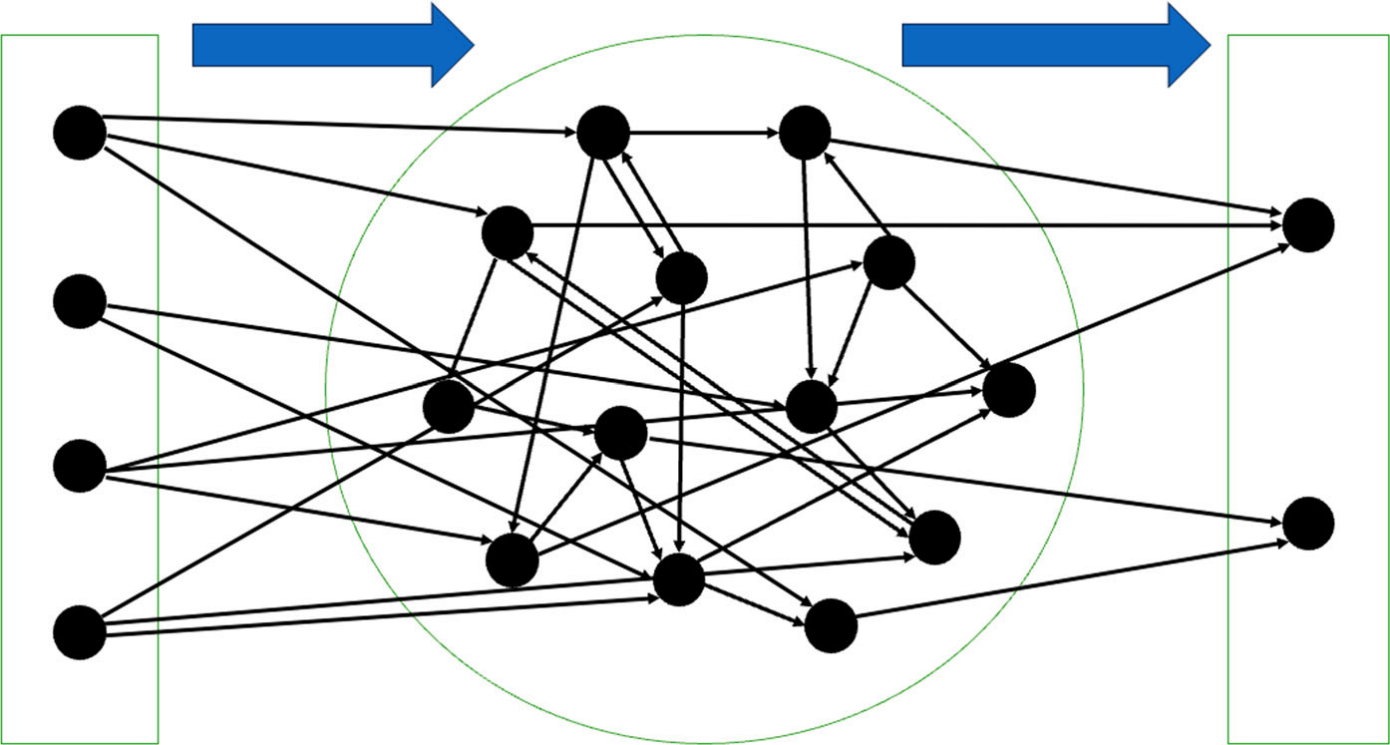
Diagram for an echo state network (ESN). From left to right are the input layer, reservoir layer and the output layer. The data flow is depicted by the blue arrow

Echo state networks (ESNs) are an instance of reservoir computing and the diagram is shown in Fig. 1. An ESN generally consists of three layers, from the left to right are: an input layer, a reservoir layer and an output layer. The weights in the input and the reservoir layer are randomly created and fixed throughout the training process and only the weights in the output layer are trainable. Here the weights in the input layer refer to the weights of the connections from the input to the reservoir layer.

First the input layer transforms the sequential data from the input layer into the reservoir layer using a randomly created input matrix. At the heart of an ESN is this fixed, large-scale recurrent reservoir of sparsely connected neurons. A distinct feature of ESNs is their echoic or fading memory property, mathematically known as the echo state property (ESP) [30, 31]. It states that an ESN has the ESP if it can forget the initial values at a rate independent of the input, given any input sequence from a compact set. This characteristic allows ESNs to efficiently capture and retain relevant information from input sequences, making them particularly adept at handling time series data. In a standard setting, there are typically a lot more neurons in the reservoir layer than in the input layer in order for the reservoir to encode the input information into a high-dimensional dynamical system using sigmoid functions. The distribution used to generate the random connections will be introduced in due course. Then the temporal representation of the input signals within the reservoir layer can therefore be used to train the output weights given the teacher signals using a standard statistical model.

After introducing the basic notions of the ESNs, we will next define the general framework of an ESN using mathematical equations. Throughout the article, we will adopt the convention for notations as below to enable the clarity and consistency for illustration: bold capital letters are used to denote matrices, bold lower case letters are used to denote vectors, and plain letters are used to denote scalars. Suppose a given longitudinal input signal has *k* features and *T* timestamps, the number of neurons inside the reservoir layer is *M* and the number of neurons in the output layer is *C*. Then the sequential data at timestamp *t* can be denoted by ${\textbf{u}}_{t}\in R^k$, the internal state in the reservoir layer by ${\textbf{x}}_{t}\in R^M$ and the output by ${\textbf{y}}_{t}\in R^C$. Here ${\textbf{u}}_{t}$ is simply the vector of the input values and ${\textbf{x}}_{t}$ is the vector of values of all neurons within the reservoir layer. The internal state is updated by

1 $$\begin{aligned} {\textbf{x}}_{t+1}= &   (1-a){\textbf{x}}_{t} + a\textbf{tanh}({\textbf{W}}_{in}{\textbf{u}}_{t+1}+{\textbf{W}}_{res}{\textbf{x}}_{t}\nonumber \\  &   +{\textbf{W}}_{back}{\textbf{y}}_{t}) \end{aligned}$$

where ${\textbf{W}}_{in}$ is the input weight matrix, ${\textbf{W}}_{res}$ is a square matrix that represents the connections of the reservoir layer, ${\textbf{W}}_{back}$ is the connections that project the output back to the reservoir and $a\in [0,1]$ is the leakage rate. However, as for the TSC tasks, the output is expected to be the predicted probabilities for distinct classes and normally only occurs at the terminal point. Thus, it may not be informative of and compatible with the temporal dynamics in the reservoir layer. As a consequence, the feedback loop will be removed in our new paradigm and the equation will be reduced to

2 $$\begin{aligned} {\textbf{x}}_{t+1} = (1-a){\textbf{x}}_{t} + a\textbf{tanh}({\textbf{W}}_{in}{\textbf{u}}_{t+1}+{\textbf{W}}_{res}{\textbf{x}}_{t}) \end{aligned}$$

Apart from the size of the reservoir layer *M*, another pivotal factor is the leakage rate *a* and it determines to what extent the internal state ${\textbf{x}}_{t}$ is susceptible to the current input ${\textbf{u}}_{t}$ and their neighbouring neurons. It has conventionally been chosen to be slightly less than 1. Moreover, the weight matrix in the reservoir layer ${\textbf{W}}_{res}$ is scaled by the spectral radius in order to balance the validity of the ESP and the performance according to [32, 33]. Lastly, the resulting output response at time *t* can be described by

3 $$\begin{aligned} {\textbf{y}}_{t} = f({\textbf{W}}_{out}[{\textbf{x}}_{t}; {\textbf{u}}_{t}]) \end{aligned}$$

where ${\textbf{W}}_{out}$ denotes the weight matrix in the output layer and *f* is an arbitrary activation function. ${\textbf{W}}_{out}$ is the only trainable part in ESNs and is normally trained by a linear classifier with input being the internal state ${\textbf{x}}_{t}$ (sometimes together with ${\textbf{u}}_{t}$) and output being the target of interest at timestamp *t*. Here we need to point out that in our new methods, the output layer will not be processed and trained as it stands which will be further clarified in due course.

### Method 1: Differential echo state networks

In this section, we will present our first method for TSC tasks, Differential Echo State Networks (Diff-ESNs) and show that it can perform efficiently on a variety of datasets with regular time series.

The ESNs are based on the fact that the current internal state ${\textbf{x}}_{t}$ is dependent on the last one ${\textbf{x}}_{t-1}$ together with the current input ${\textbf{u}}_{t}$. Let us assume that a dataset contains *N* time series samples and the observation of sample *i* at timestamp *j* can be represented by ${\textbf{u}}_{i,j}= [u^{1}_{i,j},u^{2}_{i,j},\dots ,u^{k}_{i,j}]^T$, where $i=1,\ldots ,N$ and $j=1,\dots ,n_i$. Here $n_i$ denotes the length of the sequence of sample *i* and *k* is the number of features in the dataset. Note that in our setting the first subscript denotes the sample index, the second subscript denotes the timestamp, and the superscript denotes the feature index. The outcome of patient *i* is denoted by $y_i\in \{1,2,\dots ,C\}$ where *C* is the total number of classes in this specific dataset.

One of the main obstacles that impedes the development of TSC techniques using ESNs is the invariable nature of the connections in most part of the ESN. In sequential prediction tasks, the output can be immediately linked to the input at each time step since the output is typically the prediction for the next observation ${\textbf{u}}_{i,j+1}$ given ${\textbf{u}}_{i,j}$. In TSC tasks however, the class of a sample can only be inferred after the time series reaches the terminal stage. Since the weights in the input and the reservoir layer are not adaptable in ESNs and as a result, the class information at the terminal point cannot effectively alter the dynamics in the reservoir layer on the basis of previous timestamps, an alternative route needs to be taken for the purpose of facilitating TSC while retaining the hyper-efficiency of ESNs. In this section, we will introduce a novel framework that leverages a bio-inspired neural coding method along with a hallmark of the dynamics in the reservoir layer to conduct TSC tasks.

The neural coding refers to the intricate way that the nervous system represents and processes information, specifically in the context of neurons and their activity patterns. There are several theories around neural coding mechanisms [34–36] and in spite of their controversy over validity, it seems plausible to boil the principles down to *Three S’s*: Spikes, Sparsity, Static suppression, and a brief description is given as follows.

1. Spikes: It is widely believed that the cross-talk between biological neurons is enabled by spikes (action potentials or firings). Therefore, the input and output signals of a neuron may be reckoned to be a sequence of binary outcomes such as 0, 0, 1, 0, 0, 0, 0, 0, 1, 0.
2. Sparsity: Biological neurons spend most of the time in a silent state in order to minimize unnecessary energetic costs, which makes 0 far more likely to occur than 1.
3. Static suppression: It is also known as event-driven processing in plainer language. It has been demonstrated that the neuronal systems in the sensory periphery have the tendency to stimulate neuronal responses when subject to drastic spatial or temporal change in external stimuli [37, 38].

Since ESNs are artificial neural networks that employ the sigmoid activation functions to process information, it may not be appropriate to convert the continuous observations into spikes. However, we may still take advantage of principle 2 and 3 to make our new method more biologically plausible and heuristic. Instead of using the original input sequence ${\textbf{u}}_{i,1},{\textbf{u}}_{i,2},\dots ,{\textbf{u}}_{i,n_i}$ for each sample *i*, here the sequence will be transformed into their adjacent pairwise differences such that the new observation for sample *i* at timestamp *j* can be denoted by

4 $$\begin{aligned} {\hat{\textbf{u}}}_{i,j} = {\textbf{u}}_{i,j+1} - {\textbf{u}}_{i,j}, \qquad j=1,2,\ldots ,n_{i}-1. \end{aligned}$$

**Fig. 2:**
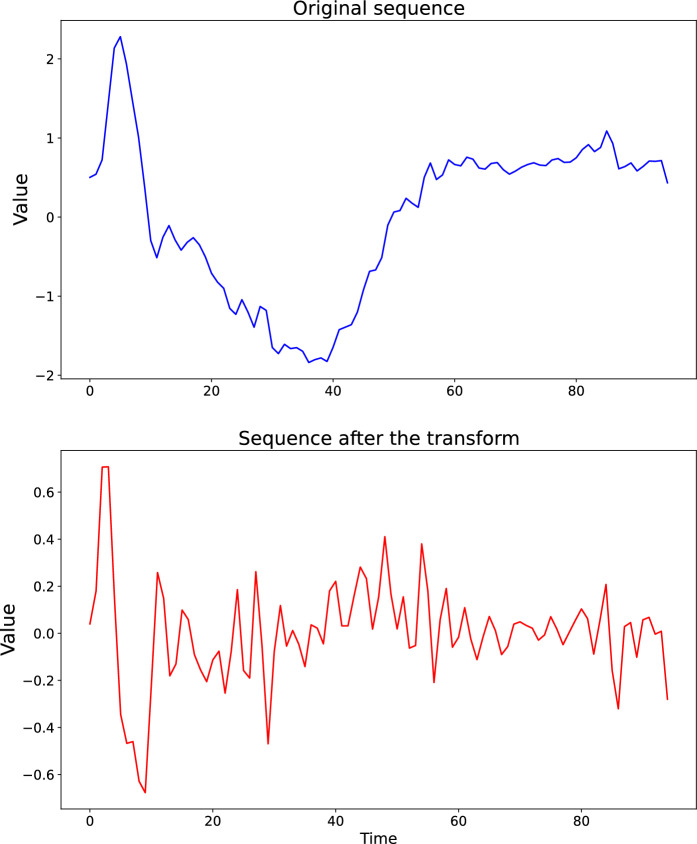
The sequence of a sample from the UCR ECG200 dataset. The upper panel exhibits the original sequence and the bottom exhibits the new sequence after being transformed by the differential operator. The x-axis indicates the timestamps and the y-axis indicates the magnitude of the observation at each timestamp

Also for the sake of conciseness, from now on, we will drop the hat on these new observations ${\hat{\textbf{u}}}_{i,j}$ and the notations will stay the same in the context of ESNs. Now the information that the neurons in the reservoir layer have to learn has been transformed from the raw observations to the sharpness of change in magnitude of the adjacent observations. Besides, after the transform, the observations at many timestamps will lie in the vicinity of 0 which corresponds to the second principal listed above. In addition to its biological plausibility, abrupt changes in time series are reckoned to entail critical information of the fate of the sample and lie at the heart of time series analysis. As a result, using the differential values as in Eq. 4 may be more informative than using the original sequence [39–41]. An example is given in Fig. 2. Here we display the sequence of a sample from the UCR ECG200 dataset before and after the aforementioned transform. As can be seen, the observations at most timestamps have been transformed in the proximity to 0 such that only the abrupt change is kept significant.

Now that we have properly defined the time series representation as the input to the ESN, we can now start formulating the model. After feeding the input sequence ${\textbf{u}}_{i,1},{\textbf{u}}_{i,2},\dots ,{\textbf{u}}_{i,n_i-1}$ for participant *i* into the ESN, the stimuli will update the internal state of the reservoir layer by

5 $$\begin{aligned} {\textbf{x}}_{i,j+1}= &   (1-a){\textbf{x}}_{i,j} + a\textbf{tanh}({\textbf{W}}_{in}{\textbf{u}}_{i,j+1}\nonumber \\  &   +{\textbf{W}}_{res}{\textbf{x}}_{i,j}) \end{aligned}$$

Note that the internal state should be reset to zero ${\textbf{x}}_{i,0}=0$ after feeding each sample so as to remove the unwelcomed dependency between samples. Then we collect all internal states for each sample *i*, ${\textbf{x}}_{i,1},{\textbf{x}}_{i,2},\dots ,{\textbf{x}}_{i,n_i-1}$ and next we need to seek a hallmark of the internal state that can be used as the features for the classifier. Here we use the variance of the sequence as the hallmark of the sample and the variance vector of sample *i* can be denoted by $\textbf{var}(i)\in R^M$. Below is a brief reasoning on the choice of this particular hallmark. From the angle of biological plausibility, there is compelling evidence that revealed variance as a signature of neural computations during decision making and that variance modulation may be present in neurons [42–44]. From the practical perspective, sequences from different classes may have distinct change levels at specific timestamps and the local level of variations can be captured by the variance to a certain extent. Thanks to the aforementioned coding method using the differential operator, the local variations can be more precisely reflected and fed into the reservoir layer. Consequently, the reservoir layer generates rich and more complex dynamics capable of better retaining short-term memory. As such, the similar level of variation at distinct timestamps can be in a way, distinguished by separate neurons in the reservoir layer. Finally, the variances of all samples in the training set will be stacked to form the input features to the classifier:

6 $$\begin{aligned} {\textbf{X}}_{train}= &   \begin{bmatrix} \textbf{var}(1)^{T}\\ \vdots \\ \textbf{var}(i)^{T}\\ \vdots \\ \textbf{var}(N_{train})^{T}\\ \end{bmatrix} \nonumber \\= &   \begin{bmatrix} \textbf{var}^{1}(1) &  \dots &  \textbf{var}^{M}(1) \\ \vdots &  \ddots &  \vdots \\ \textbf{var}^{1}(i) &  \dots &  \textbf{var}^{M}(i) \\ \vdots &  \ddots &  \vdots \\ \textbf{var}^{1}(N_{train}) &  \dots &  \textbf{var}^{M}(N_{train})\\ \end{bmatrix} \end{aligned}$$

Here ${\textbf{X}}_{train}$ can be regarded as the design matrix in the regression model. $\textbf{var}(i)$ is a column vector and the superscript *T* denotes the transpose of the vector. Similarly, the corresponding classes will be stored in a vector ${\textbf{y}}_{train}$:

7 $$\begin{aligned} {\textbf{y}}_{train} = [y_1,\dots ,y_i,\dots ,y_{N_{train}}]^T \end{aligned}$$

Then we fit ${\textbf{y}}_{train}$ to ${\textbf{X}}_{train}$ using the linear support vector machine (SVM) to train the classifier [45] as it is effective in high dimensional space, not least when the number of samples is not sufficient enough as compared to other statistical machine learning methods such as logistic regression. Lastly, the feature matrix and the classes of the test set can be defined in a similar way and we denote them by ${\textbf{X}}_{test}$ and ${\textbf{y}}_{test}$. In order to evaluate the performance of the model, we apply the trained classifier to ${\textbf{X}}_{test}$, ${\hat{\textbf{y}}}_{test} = CL({\textbf{X}}_{test})$ so that the outcomes of distinct samples can be predicted and compared with the ground truth outcomes. Here ${\hat{\textbf{y}}}_{test}$ is the predicted class assignment and *CL* is the classifier. The performance of the method will be shown in Sect. 3.1.

### Method 2: Interpolation echo state networks

As will be shown in Sect. 3.1, the Diff-ESNs have the potential to perform well when the time series data are regular. However, some datasets are more complicated than that due to the intrinsic difficulties in the data collection process. In some extreme cases, different samples (sequences) can have different numbers of observations with irregular time intervals whilst each sample may only have a handful of timestamps ($<6$).

In this work, we use ovarian cancer screening to encapsulate the context of this particular data type and highlight the importance of tackling it. Ovarian cancer is a hereditary and lethal disease that disproportionately hits women aged above 50 and causes more than 150,000 overall deaths in the UK between 2017 and 2019. It accounts for more mortalities than any other cancer arising from the female reproductive system. It is reported that the chance of a woman getting ovarian cancer is 1.3% and dying from it is 0.9% during her liftime. Whereas the 5-year survival rate is only around 40% due to late diagnosis, of which the majority of cases are diagnosed at stage III and IV, up to 90% of patients at stage I can be cured with conventional therapies, indicating the importance of early detection and intervention [46]. In order to address this long-standing health concern for ovarian cancer, various programs have been deployed worldwide which aim to discover early signs of cancer before the symptom appears when the medical intervention is more likely to be effective [47, 48]. Thus far, one of the most popular tests for ovarian cancer is the screening for tumour biomarkers. The participants have their samples taken several times in a time span of years so that their risks are well tracked and monitored. Some of them, unfortunately, will be diagnosed with cancer in the process of screening and will be transferred to medical treatment. As one can well imagine, the engagement of the participants is a highly spontaneous and independent behaviour which essentially makes the time series data exceptionally irregular. Furthermore, it is often too late for the treatment to kick in when the cancer is confirmed and as a result, early detection and prevention is also part and parcel to the wider population as well as to the optimization of the public health resources. Consequently, we will develop another method that can appropriately handle this type of datasets in the context of ESNs.

Unlike the Diff-ESNs, here the idea is to enhance the continuity of the time series such that more observations can be generated and the timestamps become more informative in the backdrop of the problem. For instance, if the biomarkers of a participant are registered at the age of 54.3, 54.9, 55.2 and 56.1, it might be of interest to construct a more continuous trajectory with observations being recorded by month given that month is a meaningful unit in clinical study. Later, we will also show that it can enable flexible early forecasting, which is strongly preferable in cancer.

**Fig. 3:**
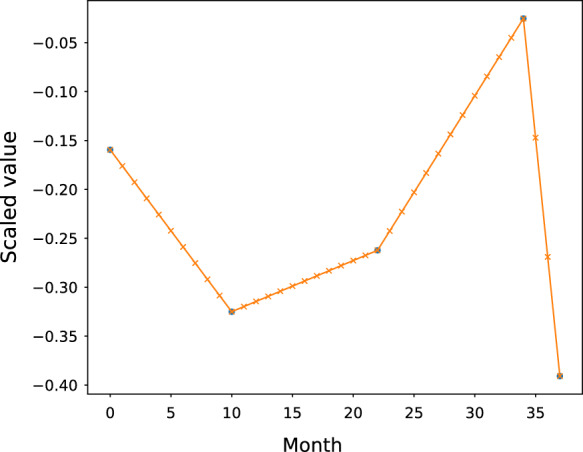
The linear interpolation of the biomarker *CA*125 of a specific participant. The x-axis stands for the month index since the start of the screening and the y-axis stands for the expression of the biomarker *CA*125. The blue dots are the original data points upon standardization and the crosses are the new data points sampled by month after interpolation

As a first step, linear interpolation is applied to the sequence of participant *i*, ${\textbf{u}}_{i,1},{\textbf{u}}_{i,2},\dots ,{\textbf{u}}_{i,n_i}$ to fit the data into a continuous curve. Then the new sequence is acquired by sampling the interpolated curve on a monthly basis since the first record of screening and again for consistency, we will adopt the same notation to denote the new sequence for each *i*. An example is given in Fig. 3. The curve exhibits the alteration in the expression of the biomarker *CA*125 from a specific participant. The blue dots signify the original data points and the crosses signify the new data points sampled by month after interpolation. A natural alternative fitting method would be the cubic spline. In our current study, the time series consists of only a handful of timestamps (fewer than 6), as compared to the whole timeline of concern. Therefore, it may not be plausible to assume the existence of nonlinear trajectory, given the existing data. One may consider fitting the data with cubic spline when the timestamps in the data are more ample and the nonlinear pattern is more effective.

Similar to the procedure in Sect. 2.2, we first collect all internal states for each sample *i*, ${\textbf{x}}_{i,1},{\textbf{x}}_{i,2},\dots ,{\textbf{x}}_{i, n_i}$. In order to prepare the internal states as the input for a linear classifier, one needs to find a way to appropriately summarize the past history, not solely the last timestamp for each participant. To this end, we first stack all internal states in the training set according to the time series for individual participants into a matrix ${\textbf{X}}_{train}$:

8 $$\begin{aligned} {\textbf{X}}_{train} = \begin{bmatrix} x^{1}_{1,1} &  \dots &  x^{M}_{1,1}\\ \vdots &  \ddots &  \vdots \\ x^{1}_{1,n_1} &  \dots &  x^{M}_{1,n_1}\\ \vdots &  \ddots &  \vdots \\ \vdots &  \ddots &  \vdots \\ x^{1}_{i,1} &  \dots &  x^{M}_{i,1}\\ \vdots &  \ddots &  \vdots \\ x^{1}_{i,n_i} &  \dots &  x^{M}_{i,n_i}\\ \vdots &  \ddots &  \vdots \\ \vdots &  \ddots &  \vdots \\ x^{1}_{N_{train},1} &  \dots &  x^{M}_{N_{train},1}\\ \vdots &  \ddots &  \vdots \\ x^{1}_{N_{train},n_{N_{train}}} &  \dots &  x^{M}_{N_{train},n_{N_{train}}} \end{bmatrix} \end{aligned}$$

And the corresponding outcomes will be stored in a vector ${\textbf{y}}_{train}$:

9 $$\begin{aligned} {\textbf{y}}_{train}= &   [y_1,\dots ,y_1,\dots \dots , y_i,\dots ,\nonumber \\  &   y_i,\dots \dots ,y_{N_{train}},\dots ,y_{N_{train}}]^T \end{aligned}$$

In a nutshell, provided that participant *i* has $n_i$ timestamps, then there will be $n_i$ entries in ${\textbf{y}}_{train}$ and $n_i$ rows in ${\textbf{X}}_{train}$. Note that here we use all timestamps to form the matrix and assume that the outcomes at all timestamps of a participant are the same. We suppose that the early observations will also provide useful information for the terminal outcome. It also aligns with our objective to enable early forecast which will be discussed in due course. However, we may not want to use all of them as training samples for two reasons:

1. The time span of a screening program is typically several years and after interpolating and sampling by month, most participants have dozens of timestamps. This may result in a substantial increase of the sample size and increase the training overheads.
2. The internal state at timestamp *j* for participant *i*, ${\textbf{x}}_{i,j}$ may already contain a certain piece of the past information of the internal state, not least those near the current timestamp *j* by virtue of the recurrent structure. Including all timestamps may increase the learning variance and fail to generalize the patterns in the datasets.

Hence, we introduce an additional parameter $\tau$, where $\tau =1,2,\dots , n_{min}$, to enable the option for users to sample the internal state ${\textbf{x}}_{i,j}$ with skips. Here $n_{min} = min\{n_{i}\}_{i=1,2,\dots ,N}$ is the minimum length of time series among all participants. Since the marker expressions at the last timestamp may be of the utmost clinical relevance, we will always retain the internal state at the last timestamp for each participant in ${\textbf{X}}_{train}$ and select rows in ${\textbf{X}}_{train}$ backwards with the skip $\tau$ such that the block for participant *i* will become

10 $$\begin{aligned} {\textbf{X}}_{i} = \begin{bmatrix} \vdots &  \ddots &  \vdots \\ x^{1}_{i,n_{i}-2\tau } &  \dots &  x^{M}_{i,n_{i}-2\tau }\\ x^{1}_{i,n_{i}-\tau } &  \dots &  x^{M}_{i,n_{i}-\tau }\\ x^{1}_{i,n_i} &  \dots &  x^{M}_{i,n_i}\\ \end{bmatrix} \end{aligned}$$

The resulting design matrix therefore becomes

11 $$\begin{aligned} {\textbf{X}}_{train} = \begin{bmatrix} {\textbf{X}}_1\\ \vdots \\ {\textbf{X}}_i \\ \vdots \\ {\textbf{X}}_{N_{train}} \end{bmatrix} \end{aligned}$$

And the corresponding subset of ${\textbf{y}}_{train}$ will be taken to form the new outcome vector and again we keep the notation unchanged, ${\textbf{y}}_{train}$. As such, enough samples can be ensured to train the classifier while taking into account the temporal dynamics and we name the approach *skip sampling*. By training the internal states that incorporate diverse lengths of history (not just the last one) of a particular participant, it also potentially enables a better generalization when seeing the longitudinal features from other participants. Then we fit ${\textbf{y}}_{train}$ to ${\textbf{X}}_{train}$ using the linear SVM to train the classifier and generate the predicted outcomes for the test set ${\hat{\textbf{y}}}_{test} = CL({\textbf{X}}_{test})$. Note that as opposed to the one shown in Sect. 2.2, here the predicted outcomes ${\hat{\textbf{y}}}_{test}$ are given in probability and the reason will be discussed at greater length later. As a result, the SVM with the probability output is implemented as a replacement [49]. Besides, at the moment, each participant still has multiple predicted outcomes and they are disparate for the same participant:

12 $$\begin{aligned} {\hat{\textbf{y}}}_{test}= &   [{\hat{y}}^{1}_1,\dots ,{\hat{y}}^{n_1}_1,\dots \dots , {\hat{y}}^{1}_i,\dots ,{\hat{y}}^{n_i}_i,\nonumber \\  &   \dots \dots ,{\hat{y}}^{1}_{N_{test}},\dots ,{\hat{y}}^{n_{N_{test}}}_{N_{test}}]^T \end{aligned}$$

One may need a single predicted outcome for each participant in order to be compared with the ground truth label. Here we select the prediction at the last time point to be the representative of the specific participant. In this way, we put more emphasis on the last time point as it not only contains arguably the most relevant information (the last screening), but also the history before that.

Early forecasting is instrumental to successful cancer treatment as it is often too late for the treatment to take effect when the cancer is confirmed. Therefore, lastly, we will demonstrate that the skip sampling approach can also enable flexible forecasting. The training process will remain the same and ${\textbf{X}}_{train}$ will be employed as the input to the classifier. Suppose that we want to make the forecast $\gamma$ months before the last record. Instead of gathering all available observations, only the observations up to the timestamp $n_i-\gamma$ will be used as the input to the classifier in the test stage:

13 $$\begin{aligned} {\textbf{X}}_{test} = \begin{bmatrix} x^{1}_{1,1} &  \dots &  x^{M}_{1,1}\\ \vdots &  \ddots &  \vdots \\ x^{1}_{1,n_1-\gamma } &  \dots &  x^{M}_{1,n_1-\gamma }\\ \vdots &  \ddots &  \vdots \\ \vdots &  \ddots &  \vdots \\ x^{1}_{i,1} &  \dots &  x^{M}_{i,1}\\ \vdots &  \ddots &  \vdots \\ x^{1}_{i,n_i-\gamma } &  \dots &  x^{M}_{i,n_i-\gamma }\\ \vdots &  \ddots &  \vdots \\ \vdots &  \ddots &  \vdots \\ x^{1}_{N_{test},1} &  \dots &  x^{M}_{N_{test},1}\\ \vdots &  \ddots &  \vdots \\ x^{1}_{N_{test},n_{N_{test}-\gamma }} &  \dots &  x^{M}_{N_{test},n_{N_{test}-\gamma }} \end{bmatrix} \end{aligned}$$

The remaining procedures stay the same and one can implement the skip sampling if needed.

## Performance

### Performance of the differential echo state networks

The performance of the method can be assessed by computing the error rate in the test set of each dataset. Namely,

14 $$\begin{aligned} ER = \frac{\sum _{i=1}^{N_{test}} I(y_i\ne {\hat{y}}_i)}{N_{test}} \end{aligned}$$

Here $N_{test}$ is the number of samples in the test set, *I* is an indicator function, $y_i$ is the ground truth class of sample *i* and ${\hat{y}}_i$ is the class category predicted by using the aforementioned trained classifier.

The UCR repository [50] is widely used as the benchmark datasets for assessing the performance of TSC algorithms. The repository contains a wide spectrum of datasets that are closely related to our daily life, including sensor, image and motion, etc. Additionally, the datasets also differ in size, length and number of classes, positioning it an ideal testbed for any newly proposed TSC methods. Each of these UCR datasets comprises a training set and a test set and the idea is to train any new model on the training set and report the error rate of the test set. In each dataset of this repository, all samples (sequences) have the same length and the lengths are all no less than 30 but can go up to several thousands for some datasets. Furthermore, all samples are recorded at the same timestamps in each dataset. In order to demonstrate the extensive application of our method, as a first step, the error rates of 33 datasets have been calculated and compared with the gold standard 1NN-DTW method. The parameters used for this study are $M=50$ and $a=0.9$. The connections in the input and the reservoir layer are fixed random matrices following the standard normal distribution. We will show that employing a small reservoir layer ($M=50$ and hence with superb efficiency) and a commonly used leakage rate *a* can lead to adequate classification accuracy. The robustness of the method will be evaluated later.

Table 1 displays the error rates generated by the Diff-ESN method, with a reference to the publicly available results using the 1NN-DTW and the LSTM-FCN from [51]. The columns from the left to the right are the name of the dataset, number of classes, training size, test size, the error rate of using the 1NN-DTW, the Diff-ESN and the LSTM-FCN. The error rates in the Diff-ESN column will be highlighted in bold colour if they outperform the 1NN-DTW method. The comparsion with the LSTM-FCN will be discussed in Sect. 4. Some dataset names are abbreviated to fit the window for better visualization. As can be seen, the Diff-ESN achieves comparable results on all these 33 datasets relative to the 1NN-DTW. Among them, the Diff-ESN outperforms the 1NN-DTW on 23 datasets and 19 of them are below 0.25. Furthermore, all these tasks can be completed on a personal computer with minimal costs ($<5$ minutes for the most training expensive dataset without even exploiting the parallel processing).

**Fig. 4:**
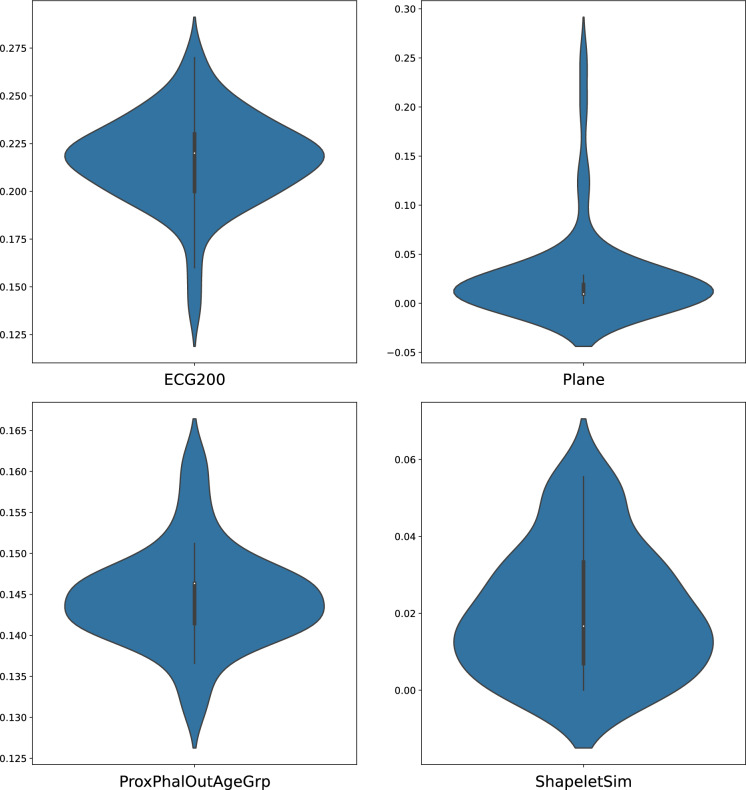
The robustness check for the Diff-ESN using the dataset ECG200, Plane, ProxPhalOutAgeGrp and ShapeletSim. Each violin plot contains 50 error rates generated by different random connections in the input and the reservoir layer. The vertical axis denotes the error rate. The means (95% confidence intervals) of their respective error rates are 0.217 (0.210$-$0.223), 0.027 (0.014$-$0.041), 0.145 (0.143$-$0.146) and 0.020 (0.016$-$0.025)

Since the connections in the input and the reservoir layer are randomly created, it is also imperative to validate the robustness of our new method. To this end, we select four datasets and have a closer look at the variation in performance subject to different connections. Figure 4 lays out the error rates of the dataset ECG200, Plane, ProxPhalOutAgeGrp and ShapeletSim produced by 50 different connections. As illustrated, the performance is barely susceptible to the change in connections. Take the dataset ECG200 for example, as a matter of fact, the reported result in Table 1 (0.24) is located in the upper side of the violin plot in the sense that the majority of the connections will give rise to a lower error rate than 0.24.

**Fig. 5:**
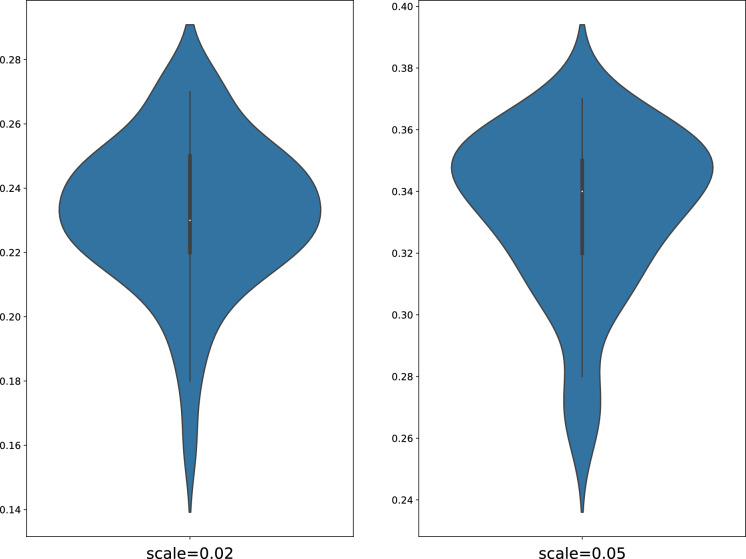
The performance of the Diff-ESN when subject to different noise levels on the test set. Each violin plot contains 50 error rates generated by different random connections in the input and the reservoir layer. The vertical axis denotes the error rate. The means (95% confidence intervals) of their respective error rates are 0.232 (0.226$-$0.239) and 0.334 (0.327$-$0.341)

Lastly, we will also have a look into the impact of the noise level on the test data on the classification accuracy. To this end, we select the dataset ECG200 and introduce two different levels of noise to the test set. In practice, we sample a noise value from the Gaussian distribution $\epsilon \sim N(0, \sigma ^2)$ at each timestamp and add to the observation value. Here $\sigma$ is the standard deviation of the Gaussian distribution. In the current study, $\sigma$ is chosen as the scale (multiplicative) of the maximum of the absolute value of the training set. Figure 5 lays out the error rates when the scale equals 0.02 and 0.05. As reflected, the robustness is largely preserved when the scale equals 0.02, but the error rates have substantially hiked when the scale increases to 0.05.

**Table 1:** Error rates of UCR datasets using the Differential ESN.

Dataset	#class	#train	#test	1NN-DTW	Diff-ESN	LSTM-FCN
BeetleFly	2	20	20	0.300	**0.250**	0
BirdChicken	2	20	20	0.250	**0.100**	0.05
CinCECGTorso	4	40	1380	0.349	**0.336**	0.155
Coffee	2	28	28	0	0.070	0
DistPhalanxTW	6	400	139	0.290	0.324	0.185
Earthquakes	2	322	139	0.258	**0.252**	0.177
ECG200	2	100	100	0.230	0.240	0.08
ECG5000	5	500	4500	0.250	**0.081**	0.055
ECGFiveDays	2	23	861	0.232	0.245	0.011
ElectricDevices	7	8926	7711	0.399	0.404	0.037
FordA	2	3601	1302	0.438	**0.120**	0.072
FordB	2	3636	810	0.406	**0.328**	0.083
Ham	2	109	105	0.533	**0.377**	0.209
Herring	2	64	64	0.469	**0.406**	0.250
ItalyPowerDemand	2	67	1029	0.050	0.076	0.038
MidPhalOutCorr	2	600	291	0.352	0.354	0.160
MidPhalanxTW	6	399	154	0.416	0.442	0.383
OSULeaf	6	200	242	0.409	**0.380**	0.004
Plane	7	105	105	/	**0.029**	0
ProxPhalOutAgeGrp	3	400	205	0.195	**0.141**	0.117
ProxPhalOutCorr	2	600	291	0.216	0.258	0.065
ProxPhalanxTW	6	400	205	0.263	**0.195**	0.167
RefrigeDevices	3	375	375	0.536	**0.528**	0.421
ShapeletSim	2	20	180	0.350	**0.039**	0.011
SmallKitchenAppl	3	375	375	0.357	**0.307**	0.184
SonyAIBORobotSurf1	2	20	601	0.275	**0.170**	0.018
SonyAIBORobotSurf2	2	27	953	0.169	**0.127**	0.022
StarLightCurves	3	1000	8236	0.093	**0.089**	0.024
ToeSegmentation1	2	40	228	0.228	**0.180**	0.013
Trace	4	100	100	0	0.020	0
TwoLeadECG	2	23	1139	0.096	**0.001**	0.001
Wafer	2	1000	6164	0.02	**0.015**	0.001
Worms	5	181	77	0.536	**0.442**	0.298

The columns from the left to the right are the name of the dataset, number of classes, training size, test size, the error rate of using 1NN-DTW, Diff-ESN and LSTM-FCN. The error rates in the Diff-ESN column will be highlighted in bold colour if they outperform the 1NN-DTW method. The LSTM-FCN column is included to evaluate the distance of our proposed to the state-of-the-art neural networks powered by backpropagtion. Some dataset names are abbreviated to fit the window. The unavailable results will be denoted by /

**Fig. 6:**
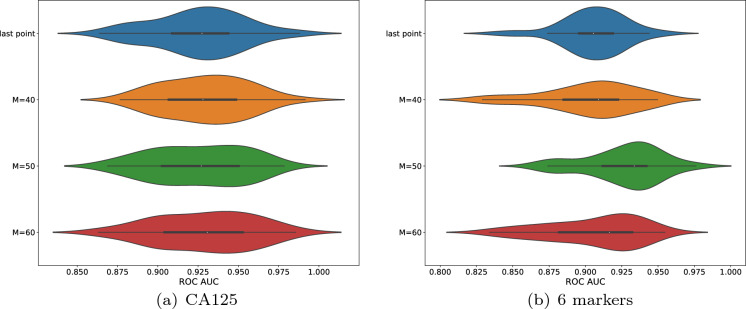
ROC AUCs of the skip sampling method. (a) shows the ROC AUC scores using only the marker CA125 and (b) shows the scores using the marker CA125, Glycodelin, HE4, MSLN25, MMP745 and CYFRA55. Each violin plot contains 50 AUC scores obtained from 50 different splits of the dataset. Each figure contains four violin plots. The first row shows the reference scores using the logistic regression model only on the last timestamp. Row 2-4 show the scores with different *M* respectively. The x-axis denotes the ROC AUC score. The means (95% confidence interval) of (a) from the top to bottom are 0.925 (0.917$-$0.933), 0.930 (0.922$-$0.937), 0.925 (0.917$-$0.933) and 0.929 (0.920$-$0.937). The means (95% confidence interval) of (b) from the top to bottom are 0.906 (0.899$-$0.912),0.902 (0.893$-$0.911), 0.926 (0.919$-$0.933) and 0.905 (0.896$-$0.914)

### Performance of the interpolation echo state networks

The error rate (or accuracy in reverse) is an intuitive measure of correctness of statistical models. The UCR benchmark datasets have been extensively studied by the wider community and the training and the test sets have been carefully pre-split for a more perspective comparison. Therefore, in Sect. 3.1, we compute the error rate of the test set and compare it with the publicly available results. However, in many other cases, it is more advantageous to generate the receiver operating characteristic curve (ROC curve) and calculate the area under the curve (ROC AUC in short form) in order to evaluate the discrimination power of the model. It comes with a few reasons. Firstly, as opposed to the error rate, the ROC curve examines all possible classification which accurately reflects the model’s response to the alteration of the threshold value. Secondly, the ROC curve is less susceptible to the imbalanced datasets. It is often the case that the clinical data are imbalanced since negative outcomes are way more likely to occur than positive outcomes. Subsequently, the error rate may still be low even if the model under-performs on the minority class and this can be substantially alleviated by the ROC curve.

In this study, the dataset that we used to assess the performance of our model is the BD dataset [52–54]. The BD dataset contains 222 patients on screening for ovarian cancer after removing those with only one timestamp. The biomarkers of interest are CA125, Glycodelin, HE4, MSLN25, MMP745 and CYFRA55, the expressions of which have been standardized before fitting any machine learning models. Among them, CA125 is a protein that has been regarded as the primary marker for ovarian cancer and the elevated levels of CA125 can be associated with certain conditions [55, 56]. The recorded times of these marker expressions are also included in the dataset which enables the interpolation of the time series.

With a view to evaluating the performance of our method, the BD dataset is split 50-50 randomly in that half of the dataset is used for training and the other half for testing. Considering that the BD dataset is a new and relatively small dataset, the robustness of the model in regard to the dataset needs to be checked carefully. With this goal in mind, 50 different splits of the dataset will be studied so as to gain a more comprehensive view of the model. Additionally, multiple values of the number of the neurons in the reservoir layer, *M*, will also be explored since changing the value of *M* will not only examine this important parameter, but also alter the connections in the input and the reservoir layer so that more random connections can be inspected at the same time.

Figure 6 displays the ROC AUC scores using the skip sampling method with $\tau =4$. Here $\tau$ is a parameter of the user’s choice but we recommend using $\tau =3,4,5,6$. On the one hand, from the biological viewpoint, many cancer types double in size every few months on average (e.g., about every 6 months for breast cancer and 4-5 months for lung cancer); on the other hand, one may not want to sample too many points as it will increase the training cost. The recurrent structure in the reservoir layer also allows the ensemble to retain the past information in the current state to some extent. Figure 6a displays the case where only CA125 is used as the input and b where all 6 markers are included. Each figure contains 4 violin plots and each violin plot exhibits 50 AUC scores that correspond to 50 different splits of the training and the test set. In order to better evaluate the discrimination power of our time series based method, a comparison has also been made with the scores using only the last time point. In the latter case, only the last recorded marker expressions are selected for each participant and the logistic regression model is fitted to the training set and the outcomes (in probability) of the participants are predicted in the test set accordingly. The first row of each figure shows the AUC scores obtained by using the aforementioned logistic regression model as a baseline result. Row 2-4 show the AUC scores obtained by the skip sampling method with $M=40, 50, 60$, respectively. As can be seen, most splits give rise to a consistently high ROC AUC score ($>0.9$), irrespective of the markers involved. In the case where only CA125 has been used, $M=40$ and $M=60$ yield a slightly better overall performance than the baseline while $M=50$ also reaches a comparable level (and arguably better in some aspects). In the case of the 6 markers, $M=40$, $M=50$ and $M=60$ all give rise to a comparable overall performance and among them, the improvement of $M=50$ on the baseline result is highly significant.

**Fig. 7:**
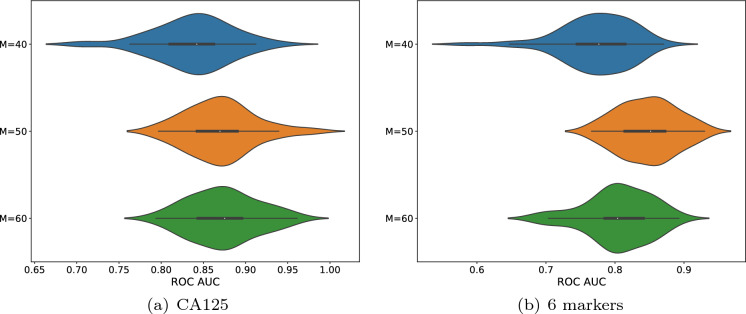
ROC AUCs of the 6-month forecast using the skip sampling method. (a) shows the ROC AUC scores using only the marker CA125 and (b) shows the scores using the marker CA125, Glycodelin, HE4, MSLN25, MMP745 and CYFRA55. Each violin plot contains 50 AUC scores obtained from 50 different splits of the dataset. Each figure contains three violin plots and they show the scores with different *M* respectively. The x-axis denotes the ROC AUC score. The means (95% confidence interval) of (a) from the top to bottom are 0.837 (0.824$-$0.851), 0.870 (0.859$-$0.881) and 0.873 (0.862$-$0.885). The means (95% confidence interval) of (b) from the top to bottom are 0.772 (0.757$-$0.787), 0.847 (0.835$-$0.858) and 0.804 (0.791$-$0.818)

Finally, the performance of the 6-month forecast is presented in Fig. 7 using the skip sampling approach. This time, only the participants with at least three timestamps will be included to ensure the existence of the 6-month window. As shown, most splits give rise to a reasonably high ROC AUC score ($>0.75$) and the majority of them lie above 0.8, which demonstrates a robust forecasting power of our method.

The reason that we want to assess the performance of different parameters (*M*) is that, as compared to using the cross validation and selecting the optimal parameter from the training set, it allows for a better inspection of the robustness subject to different parameters, which is reckoned crucially important for any new methodologies.

## Conclusion and discussion

In this work, we established two ESN methodologies, Diff-ESN and Interp-ESN, that can effectively address regular and irregular TSC tasks, respectively. The Diff-ESN was tested on the standard benchmark datasets and the Interp-ESN was tested on a recent cancer screening dataset. Both of them exhibit desirable accuracy as well as tremendous efficiency.

We demonstrated that the Diff-ESN method has attained comparable performance with the classic 1NN-DTW method and resulted in notable improvement on error rate on several datasets, as shown in Table 1. It is also worth reiterating again that our methods took the inspiration from the means that the brain receives and processes information and leveraged the simplest possible structure in RC, which differs from many other aforementioned ESN-based TSC methods. The Interp-ESN method has been designed to tackle the famously hard irregular time series such as cancer screening data where forecasting on a regular basis is desired. All cases that we considered as in Fig. 6 achieve at least no worse overall performance than the baseline using the logistic regression on the last recorded time point. Some parameters have given rise to a significant increase in performance in terms of the aspects such as mean, median, confidence interval and inter-quartile. Most importantly, the method entails a flexible forecasting option that empowers the prediction of the outcome at any month in advance and attains high ROC AUC scores with a 6-month forecast. To the best of our knowledge, the existing ESN-based methods do not emphasize handling irregularity or forecasting.

With the contribution being said, our model comes with several limitations and some further considerations can be given in the future. In particular, one may realize that we actually proposed two separate methods based on the ESNs to tackle regular and irregular data individually. First of all, the Diff-ESN may not be an appropriate approach for the irregular time series such as the cancer screening data. It is because that the interpolated sequence only yields a predicted coarse trajectory and the differential operator will only compute inaccurate difference between two adjacent timestamps. Therefore, the hallmark of the trajectory that we chose, variance, may not be as predictive as when applied to the aforementioned regular data. One may also note that each time series sequence in cancer screening data only contains a handful of observations and the dynamics are relatively simple. As a result, the variation in the trajectory may not be as informative as the values themselves at some key timestamps when it comes to terminal prediction. The same problem may also arise when the variation in observation value is not the main source of differentiation among different class groups. Secondly, even though the Interp-ESN, not least the skip sampling approach, may be deemed as a more generic method, it comes with inevitable inconvenience when being applied to the regular time series data such as those listed in Table 1. The most prominent one is the pre-specified sampling step and its trade-off with the computational efficiency. Even a sizable step size can still incur a relative high computational cost when the time series sequence is exceptionally long and there is no guarantee that the selected sampling step is informative in any possible way. In addition, to begin with, the interpolation has already increased the intrinsic noise in the trajectory (since most observations in the trajectory are unknown). Hence, the method may not remain robust if the original data are noisy. Lastly, there still exists a significant gap between the performance of our method reported in Table 1 and the state-of-the-art LSTM neural networks shown in [51] for most of the datasets. The last column of Table 1 outlines the error rates obtained using the LSTM-FCN [57]. Yet, the deep neural networks require the training of an astronomical amount of parameters through back-propagation whereas our method is free of back-propagation and the training can be completed with a negligible energy cost. For a standard ESN, the computational complexity for a time series sequence is ${\mathcal {O}}(T(Mk+M^2)$. For a simple LSTM layer (only with the input, forget and output gates), the computational complexity for a time series sequence is ${\mathcal {O}}(2TM + 6T(Mk+M^2))$, with the forward and the backward pass combined. Here *T* is the length of the sequence, *M* is the number of neurons in the reservoir layer (or hidden units) and *k* is the dimension of the input. As one can easily tell, the computation cost can enormously increase when *T* is large for an LSTM layer. Moreover, as in [57], in order to attain the reported level of accuracy, one normally needs a deep network and sometimes it comes in conjunction with additional architectures such as convolutional networks as outlined in the paper. This will increase the computational complexity by an order of 10 or even more.

The future direction may lie in a more sensible trade-off between the classification accuracy and the energy consumption. One might consider introducing a light training algorithm for the weights in the reservoir layer as well as the output layer at each time step. This is also in compliance with the observation in Figs. 4, 6 and 7 that some random connections lead to better performance than others. However, it is not very clear how to fix the computational complexity and whether the convergence will be guaranteed or not. Another direction is to learn the success of LSTM networks and attention transformers when it comes to capturing long-term dependency. This could result in the consideration of designing multiple reservoir blocks with various time scales.

## Data Availability

The code can be made available on request.
